# 

**Published:** 1889-09

**Authors:** 


### SPECIAL NOTICE TO NEW SUBSCRIBERS.

WE offer the JOURNAL from now on to January free to all persons who will send in the subscription price, $2.50, for the year 1890, before the 15th of October. We make this unprecedented offer in order to increase our subscription-list, well knowing that when a subscriber's name is once on our books, he will not drop out. This offer is only made to persons who have not been subscribers. Please take the trouble to tell your friends of the above offer.

